# β2 Adrenergic Regulation of the Phagocytic and Microbicide Capacity of Macrophages from Obese and Lean Mice: Effects of Exercise

**DOI:** 10.3390/nu11112721

**Published:** 2019-11-09

**Authors:** Leticia Martín-Cordero, Isabel Gálvez, María Dolores Hinchado, Eduardo Ortega

**Affiliations:** 1 Grupo de Investigación en Inmunofisiología, Departamento de Enfermería, Centro Universitario de Plasencia, Universidad de Extremadura, 10600 Plasencia, Spain; leticiamartin@unex.es; 2 Instituto Universitario de Investigación Biosanitaria de Extremadura (INUBE), 06071 Badajoz, Spain; igalvez@unex.es (I.G.); mhinsan@unex.es (M.D.H.); 3 Grupo de Investigación en Inmunofisiología, Departamento de Fisiología, Facultad de Ciencias, Universidad de Extremadura, 06071 Badajoz, Spain

**Keywords:** β2 adrenoreceptors, macrophages, obesity, exercise, innate response, inflammation, phagocytosis, microbicide activity

## Abstract

Macrophages are crucial in the inflammation associated with obesity. Exercise is the main non-pharmacological strategy against obesity, not only for improving metabolic impairment, but also because of its anti-inflammatory effects, particularly those mediated by β2 adrenergic receptors (β2-AR). Nevertheless, these anti-inflammatory effects could immunocompromise the innate response against pathogen challenge. Thus, the objective of this work was to evaluate the effect of obesity, and of exercise in this condition, on the β2 adrenergic regulation of the innate function of macrophages. High fat diet-induced obese C57BL/6J mice were used to evaluate the effects of acute and regular exercise on the phagocytic and microbicide capacities of peritoneal macrophages. Selective β2-AR agonist terbutaline (1 µM) decreased the phagocytic and microbicide activities of macrophages from control lean and obese sedentary animals. While acute exercise did not modify the inhibitory capacity of terbutaline, regular exercise abolished this inhibitory effect. These effects cannot be explained only by changes in the surface expression of β2-AR. In conclusion, (1) obesity does not alter the β2-AR-mediated decrease of the innate response of macrophages and (2) regular exercise can revert the inhibitory effect of terbutaline on the phagocytic activity of macrophages, although obesity seems to hinder this immunophysiological adaptation.

## 1. Introduction

Inflammation is part of the innate immune response mechanisms against pathogens. An appropriate and satisfactory inflammatory response results in the elimination of the infectious agents followed by a resolution and repair phase, which is mediated primarily by resident and recruited macrophages [1]. Phagocytosis and the microbicide activity of macrophages is the “first hurdle” confronting infectious diseases in the body, but it is also a key component in regulating innate and adaptive immune function [2].

It is well-known that both obesity [3] and exercise [4,5] affect the innate/inflammatory immune response mediated by macrophages, and that neuroimmunomodulation participates in these responses mainly through catecholamines [6,7]. Catecholamines secreted by the sympathetic nervous system (SNS) and the adrenal glands are important immunoregulatory molecules of macrophages through both α- and β-adrenergic receptors (AR), and adrenergic agonists interfere with their innate/inflammatory response [8,9]. Indeed, in addition to obesity-associated alterations of the innate/inflammatory response [7,9,10,11], noradrenergic dysfunction, including over-activity of the SNS, is today also recognized as a characteristic of obesity and metabolic syndrome, contributing to the pathophysiology and clinical prognosis [12,13]. It had been shown that the obese Zucker rat (a common animal model of metabolic syndrome) presents a dysregulation in the feedback mechanism between inflammatory cytokines and noradrenaline (NA), and that the regulation of the macrophage-derived cytokines by NA and phagocytic activity is defective in these animals. This could contribute to the low-grade inflammation and greater susceptibility to infections associated with obesity [7,14,15]. Changes in the recruitment and activation of macrophages in the adipose tissue also contribute fundamentally to the regulation of metabolic homeostasis, and a pathogenic or protective role of macrophages depending on their profile in experimental models of obesity has also been suggested [16,17].

In this context, it is currently accepted that most of the important beneficial effects of exercise in obesity are exerted through its anti-inflammatory effects, mediated through the decrease in the percentage of cells with inflammatory profile and the increase in NA levels as an anti-inflammatory mediator [18]. Regular physical exercise, therefore, can generate anti-inflammatory benefits by switching the inflammatory phenotype of monocytes and macrophages in obese individuals [18]. However, and because the inflammatory response constitutes an intrinsic part of the innate immune response against pathogens, the anti-inflammatory effects of physical exercise, if not well regulated, could induce immunocompromised states in obese individuals, further increasing susceptibility to infections [19]. In this sense, it is very important to avoid inadequate or “sterile” inflammation, without immunocompromising the innate responses. Thus, the term "bioregulatory effect of exercise" is defined to be one that reduces or prevents any excessive effect of inflammatory mediators and stimulates (or at least does not impair) the innate defenses (particularly phagocytosis and microbicide activities) against pathogens; also inducing suitable transitions from a pro-inflammatory to an anti-inflammatory profile of macrophages, thus generating immunophysiological adaptations through an optimal balance between pro- and anti-inflammatory responses [5]. These effects are mediated, at least partially, by catecholamines, with NA playing an especially important role [5].

It is well-established that NA is involved in regulating most of the mechanisms of the immune response, including the innate response and the systemic and local release of inflammatory cytokines [3,7,20,21]. The NA-induced inhibition of the production of pro-inflammatory cytokines and stimulation of anti-inflammatory ones by immune cells formed the basis for accepting the anti-inflammatory effects of catecholamines. This effect is mainly mediated by β2-adrenoceptors (β2-AR), which is present in the membrane of macrophages and other immune cells [22]. Thus, β2-AR stimulation and NA decrease the synthesis and release of inflammatory mediators from activated macrophages [14,23,24,25], and induce the release of anti-inflammatory cytokines [26]. Nevertheless, these effects can be altered in obesity and by exercise, particularly in this pathophysiological condition [9]. In this context, we recently reported an anti-inflammatory effect of β2-adrenergic stimulation on circulating monocytes with a pro-inflammatory state in high-fat diet-induced obesity in C57BL/6J mice. However, β2-adrenergic stimulation in monocytes was anti-inflammatory only in obese animals, which presented a pro-inflammatory state at baseline, but not in healthy lean ones [8], confirming that obesity can affect the adrenergic regulation of innate cells. However, the β2-adrenergic regulation of the innate immune response (phagocytosis and microbicide capacity) of macrophages has been very scarcely studied; and nothing has been reported with respect to the influence of obesity in this regulation. Also, the role of physical activity on the mechanisms underlying this regulation in obesity are still not well understood. Moreover, taking into account that the influence of obesity on this β2-adrenergic regulation could also be modulated by exercise (a non-pharmacological strategy for obesity) [9], and that a β2-adrenergic inhibition of the inflammatory response could immunocompromise the phagocytic response of macrophages, the objective of the present investigation was to determine the effect of obesity and of exercise in this pathophysiological condition on the β2-adrenergic regulation of the phagocytic and microbicide capacity of peritoneal macrophages from C57BL/6J mice.

## 2. Methods

### 2.1. Animals and Experimental Design

To generate mice used in this study, C57BL/6J mice were housed and bred in the animal facilities of the University of Extremadura from stock originally obtained from Envigo (Huntingdon, United Kingdom). At eight weeks of age, 34 mice were randomly allocated to one of two diets until sacrifice 18 weeks later. In order to obtain an experimental model of obesity, one group (*n* = 17) (obese group) was placed on a high-fat diet (HFD) (260HF diet; SAFE, Augy, France) containing 36% fat (58.8% of the energy from fat). This diet is ideal for studying obesity and its complications in mice. The other group (*n* = 17) constituted the healthy control group (lean group) and was placed on standard laboratory rodent chow (SD) (A04 diet; SAFE, Augy, France), containing 3.1% fat (8.4% of the energy from fat).

Mice were housed individually in cages with free access to food and water throughout the study. The cages were kept in a temperature- and humidity-controlled room (temperature, 22 ± 1 °C; humidity, 60% ± 5%) and exposed to a 12 h light/12 h dark cycle (light period 23:00–11:00; dark period 11:00–23:00). Weight and food consumption measurements started in the first week of the protocol (eight weeks of age) and continued weekly for the entire lifespan of each mouse. Food consumption was determined by weighing the total amount of food given at the start of each week and then subtracting by the amount of food remaining at the end of the week.

After 10 weeks of diet protocol, at approximately 4.4 months of age, a group of obese mice (*n* = 6) and a group of lean mice (*n* = 6) were subjected to a habitual exercise protocol (trained group) for eight weeks. In addition, after 18 weeks of diet protocol, another group of obese mice (*n* = 5) and of lean mice (*n* = 5) were subjected to an acute bout of exercise (acute exercise group) immediately before sacrifice. The rest of the animals, both lean (*n* = 6) and obese (*n* = 6), did not perform any kind of physical exercise (sedentary group).

**Figure 1:**
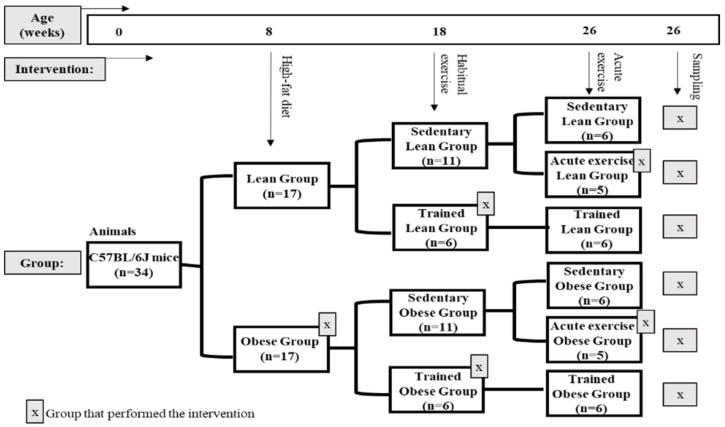
Schematic diagram of the experimental design of the study.

After 18 weeks of diet protocol, at approximately 6.5 months of age, after a 12 hour fast for all the groups and 72 hours of rest for the trained groups, peritoneal suspension and blood samples were collected from anaesthetized animals. All of the evaluated parameters were determined in each animal (Figure 1).

The study was approved by the Bioethics Committee for Animal Experimentation of the University of Extremadura (registry number 115/2015), in accordance with the national and European legislation for the protection of animals used for research.

Figure 1 shows a schematic diagram of the experimental design of the study.

### 2.2. Exercise

The habitual exercise protocol was performed three days per week, for eight weeks, always at the same time and in the active period of animals (dark period, 11:00–23:00), starting at approximately 12:00. The habitual exercise training consisted of running on a treadmill (model 800, IITC Life Science Inc., Los Angeles, CA, USA) with no slope, based on adaptation, progression, and maintenance phases in intensity and duration from 10 m/min for 10 min in the first week to values close to 18 m/min for 45 min in the last week. This protocol of regular exercise is accepted to induce physiological adaptations in mice [27,28]. Mice were sacrificed 72 hours after the last training in order to avoid the evaluation of the acute effects of exercise.

The bout of acute exercise was performed in the active period of animals (dark 11:00–23:00), starting at approximately 12:00, and consisted of running on the treadmill for 5 min at 10 m/min followed by a progressive increase to 16 m/min for 35 min, with no slope. Animals were sacrificed immediately after the bout of exercise.

### 2.3. Anaesthesia 

Mice were gas anaesthetized with isoflurane, by standard procedures (with a starting dose of 3%–5% isoflurane, and a maintenance dose of 1.5%–3% isoflurane). Then, whole blood was drawn from live, anaesthetized animals by cardiac puncture using heparinized syringes.

### 2.4. Collection of Biological Samples, Cell Culture, and Incubation

Blood: blood was extracted from live animals, drawn from the heart by cardiac puncture using heparinized syringes. A total of 50 µl of whole blood was taken for the direct determination of fasting glucose levels and lipid profile (total cholesterol (TC), high-density lipoprotein cholesterol (HDL-C), calculated low-density lipoprotein cholesterol (cLDL-C), triglycerides (TG)) using the portable analytical device LUX^®^ (Biochemical Systems International Srl, Arezzo, Italy). Glucose levels were determined using reagent strips based on electrochemical methods (glucose oxidase method), while the results of the lipids test were based on the reading of the light reflected from the test strip (reflectometry method).

Peritoneal cells: Peritoneal suspension was extracted from live animals. Abdominal skin was removed and 4 mL of phosphate buffered saline (PBS) were injected into the peritoneal cavity. Then, the abdomen was massaged, and approximately 3 mL of fluid were removed and deposited in polypropylene tubes. Peritoneal suspension was centrifuged, counted, and diluted to 10^6^ cells/mL in RPMI 1640 complete medium (L-glutamine and penicillin–streptomycin) (Thermo Fisher Scientific, Waltham, MA, USA) except fetal bovine serum (FBS) and distributed in 24-well plates. Culture cells were incubated in the presence or absence of the selective β2-AR agonist terbutaline (1 μM) (Sigma-Aldrich, St. Louis, MO, USA) with or without the β2-selective blocker butaxamine (5 μM) (Sigma-Aldrich MerkMillipore, Germany) (to check the effect of terbutaline). Plates were incubated for five hours at 37 °C in a 5% CO_2_ incubator.

Then, culture cells were centrifuged at 300 *g* for 10 min. Supernatants were discarded and pellets were incubated in 600 µL of staining buffer, consisting of cold PBS solution with 0.5% bovine serum albumin (BSA) (Thermo Fisher Scientific, Waltham, MA, USA), and 2 mM ethylenediaminetetraacetic acid (EDTA) (Thermo Fisher Scientific, Waltham, MA, USA), to obtain attached cells for antibody staining, or PBS solution with 2% FBS for phagocytosis and oxidative burst assays.

### 2.5. Phagocytic and Oxidative Burst Assays via Flow Cytometer

The phagocytosis and microbicide capacity of opsonised bacteria were assessed in macrophages from the peritoneal cavity through a flow cytometry analysis. This quantitative technique enables a very accurate determination of the ability of macrophages to ingest bacteria and produce superoxide anion (O2-, indicative of oxygen-dependent microbicide capacity), as measured using the mean fluorescence intensity (mfi) of active phagocytic cells.

First, opsonized bacteria (*Escherichia Coli*) were fixed in paraformaldehyde (PFA) (1%) and stained with FITC (fluorescein isothiocyanate) at a final concentration of 30 μg/m.

Cellular suspension was incubated (for 30 minutes at 37 °C in the dark with shaking) with *E.coli*-FITC, Hoechst 33342 (10μg/ml), hydroethidine (HE) (10μM) (a specific probe used to detect the intracellular production of superoxide anions by NADPH oxidase), and To-Pro-3 (0.1 μM) diluted in PBS with 2% FBS.

Samples were analysed by MACSQuant 10 flow cytometer (Miltenyi Biotech, Germany). Data were analysed by MACSQuantify (Miltenyi Biotech) and Flowlogic (Innivai, Australia) software. The results are expressed as the percentage of ‘phagocytic macrophages’ (macrophages CFSE+) or ‘microbicidal macrophages’ (macrophages HE+), and as measurements of the phagocytic or oxidative activity (mean fluorescence intensity, mfi).

### 2.6. β2-Adrenergic Receptor Expression Assays via Flow Cytometer

For the evaluation of the surface expression of β2-AR in peritoneal macrophages, cell suspension at 1 × 10^6^ cells/mL was used. Cells were diluted in 600 μL of staining buffer plus 750 μL of Inside Fix reagent from Inside Stain Kit (Miltenyi Biotec, Bergisch Gladbach, Germany, Germany) for fixation of cells for intracellular staining during 25 minutes at room temperature in darkness and agitation. After that, cells were spun down and washed in 300 μL of staining buffer, and kept overnight at 4 °C. Then, cells were spun down again and re-suspended in 50 µL of Inside Perm reagent from Inside Stain Kit. The aliquots were incubated for 20 minutes at room temperature and in the dark with the conjugated antibody ADRB2 (Polyclonal Antibody, Alexa Fluor 647 Conjugated, Bioss Antibodies, USA) (25ng/ml) for the evaluation of membrane expression of β2-AR. Dilution of the antibody was established after titration. Next, all the samples were washed and fixed for the later reading and acquisition of data by flow cytometry.

### 2.7. Flow Cytometry Assays

Flow cytometry is a technique that allows the phenotypic evaluation of cells by basically analysing three parameters: size, internal complexity, and relative intensity of fluorescence emitted by fluorochromes bound to them. In this work, after the incubation process and subsequent cellular labelling with the conjugated antibody and cellular fixation, the samples were subjected to analysis by a flow cytometer. Data were processed using the CytExpert software (Beckman Coulter Life Sciences, Indianapolis, IN, USA) and analysed using macrophage population gated by FSC/SSC parameters.

### 2.8. Statistical Analysis

In the present work, we evaluate the effect of exercise on the β2-adrenergic regulation (through selective β2-AR agonist terbutaline) of the phagocytic and microbicide capacity of peritoneal macrophages from obese and lean C57BL/6J mice.

Values are expressed as mean ± standard error of the mean (SEM). The variables were normally distributed (tested by the Kolmogorov–Smirnov normality test). One-way analysis of variance (ANOVA) test was used for comparisons among multiple groups. Student’s *t*-test was used for comparisons between the pairs of groups (paired or non-paired samples). The minimum significance level was set at *p* < 0.05.

## 3. Results

### 3.1. Body Weight, Dietary Intake, and Fasting Blood Glucose and Lipid Profile. Effect of Exercise

As expected, at the end of the high-fat diet (HFD) protocol, the obese group presented significantly higher body weight than the animals fed standard chow, even with HFD-animals ingesting less food than the control group. This greater weight increase even with a lower food intake can be explained by the fact that daily energy intake was higher in obese mice than in lean mice, as a result of the higher energy value of the HFD. The obese group presented significantly higher levels of glucose, triglycerides, total cholesterol, HDL-C, and cLDL-C than the lean group. These values are shown in Table 1, and confirm that this model of HFD protocol is good to induce an experimental model of obesity and obesity-associated metabolic dysregulation in C57BL/6J mice.

Lean and obese mice presented lower blood glucose levels immediately after performing an acute bout of exercise, which is because of the elevated glucose uptake during exercise. However, and although a trend towards lower fasting glucose levels was observed, neither lean nor obese animals performing the program of habitual exercise showed significant changes in glucose levels (which were measured 72 hours after the last training). Fasting levels of HDL-C increased and cLDL-C decreased in both groups of trained animals, an effect particularly relevant in the obese group. Triglycerides concentration levels were also significantly lower in the trained groups compared with the sedentary groups (Table 1). Thus, it is noteworthy that the lipid profile improved in both lean and obese animals after habitual exercise, but not after acute exercise, as reflected in HDL-C, cLDL-c, and triglycerides values. Unfortunately, it was not possible to determine cholesterol values after acute exercise in lean animals because they were below detection limits. These results confirm that the protocol of regular exercise is optimal for evaluating exercise-induced physiological and metabolic adaptations in HFD obese mice.

### 3.2. Effect of β2-Adrenergic Activation by Terbutaline on the Phagocytic and Microbicide Capacities of Peritoneal Macrophages from Lean and Obese Mice

**Figure 2:**
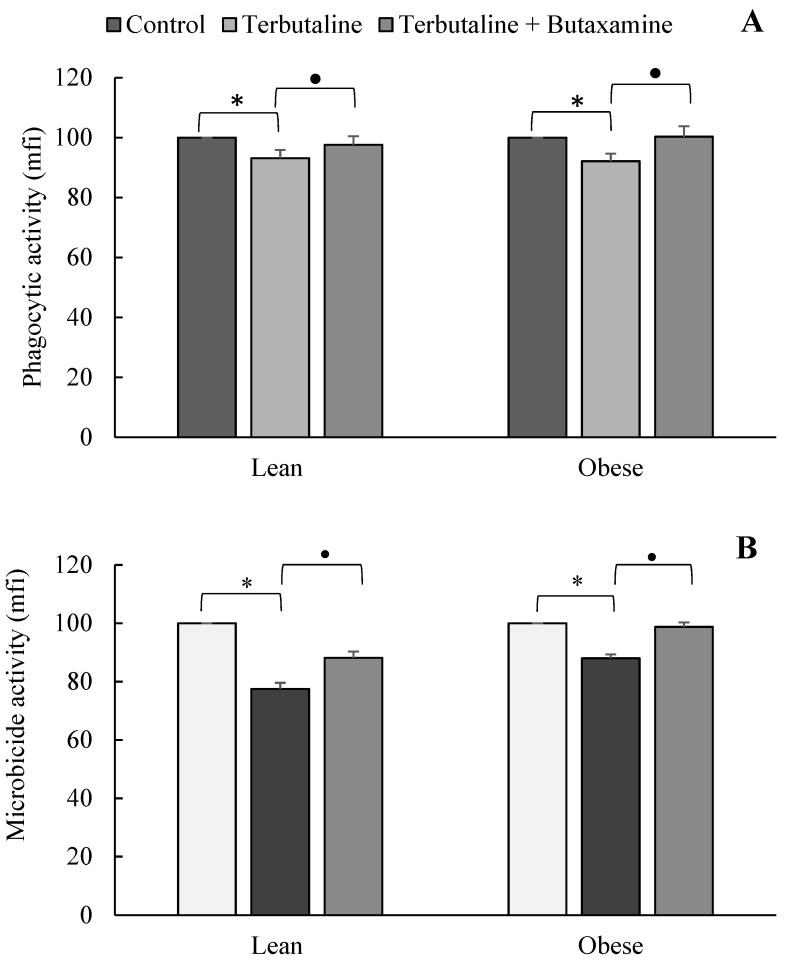
Effect of the β2-adrenergic agonist terbutaline and β2-adrenergic antagonist butaxamine on the phagocytic activity (mean fluorescence intensity, mfi) (**A**) and microbicide activity (mfi) (**B**) of peritoneal macrophages from lean and obese mice, expressed as the percentage change from baseline (giving “100” to control values in the absence of terbutaline and butaxamine). Columns represent the mean ± SEM of independent assays performed in duplicate. * *p* < 0.05 with respect to the corresponding control value; • *p* < 0.05 with respect to the corresponding terbutaline value.

Figure 2 shows the results corresponding to the effect of terbutaline on the phagocytic and microbicide activities of peritoneal macrophages in lean and obese animals. β2-adrenergic stimulation by terbutaline significantly decreased (*p* < 0.05) the phagocytic (Figure 2A) and microbicide activities (Figure 2B) of macrophages, and the presence of butaxamine (β2-AR blocker) abolished these effects. The results and statistical study of the effect of terbutaline on the percentage of macrophages presenting phagocytic and microbicide activities are shown in Table 2.

### 3.3. Effect of Exercise in the β2-Adrenergic Regulation of the Phagocytic Capacity of Peritoneal Macrophages from Obese and Lean Mice

**Figure 3:**
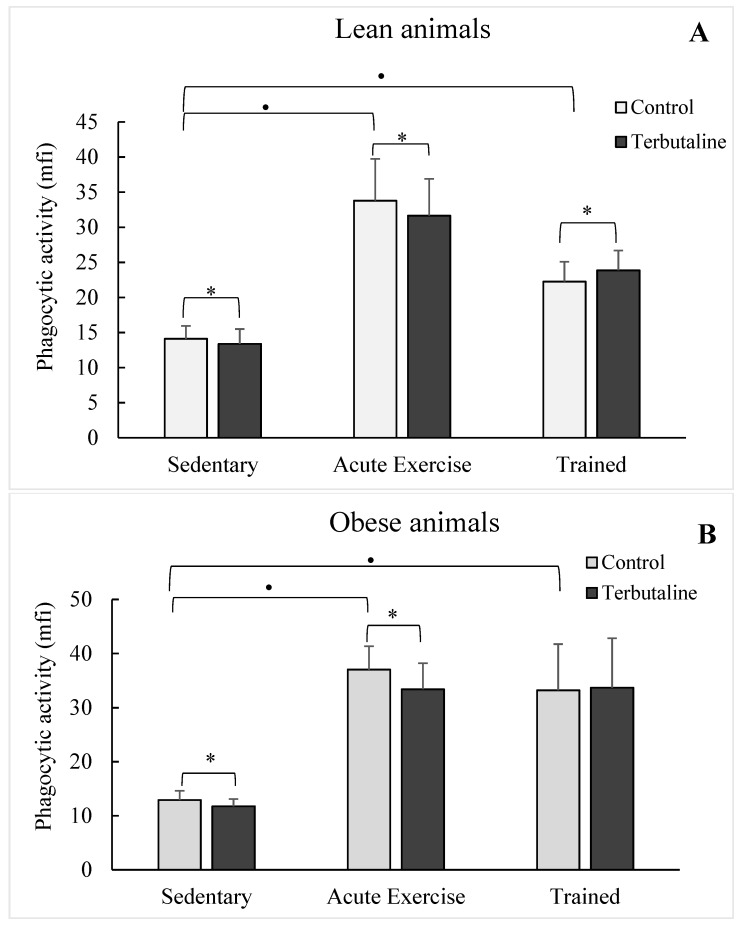

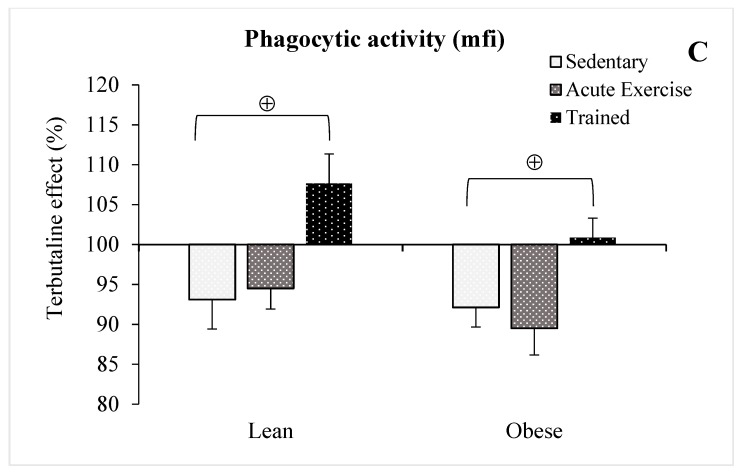
Effect of the β2-adrenergic agonist terbutaline on the phagocytic activity of peritoneal macrophages from lean (**A**) and obese (**B**) mice (sedentary, acute exercise, and trained mice). Effect of terbutaline expressed as percentage change from baseline (**C**). Columns represent the mean ± SEM of independent assays performed in duplicate. * *p* < 0.05 with respect to the corresponding control value, in the absence of terbutaline; • *p* < 0.05 with respect to the sedentary control group; ⊕ *p* < 0.05 with respect to the corresponding sedentary group.

**Figure 4:**
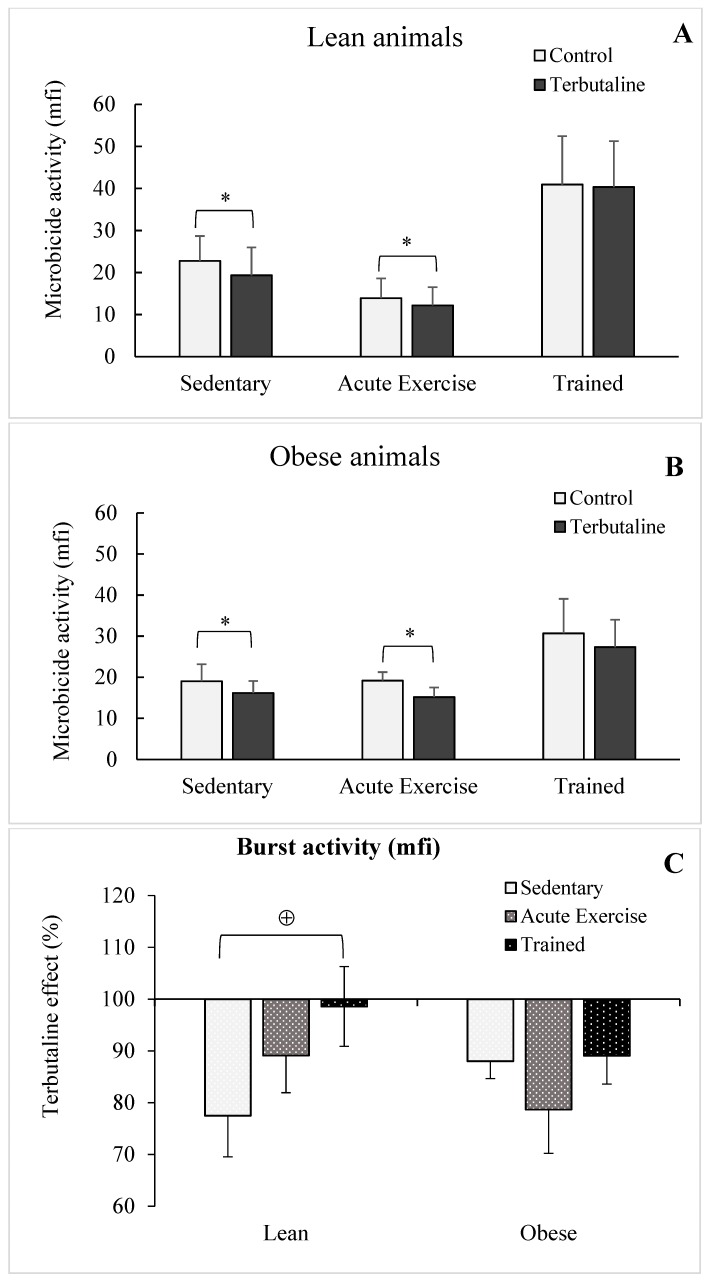
Effect of the β2-adrenergic agonist terbutaline on the microbicide activity of peritoneal macrophages from lean (**A**) and obese (**B**) mice (sedentary, acute exercise, and trained mice). Effect of terbutaline expressed as percentage change from the baseline (**C**). Columns represent the mean ± SEM of independent assays performed in duplicate. * *p* < 0.05 with respect to the corresponding control value, in the absence of terbutaline; ⊕ *p* < 0.05 with respect to the corresponding sedentary group.

Macrophages isolated from acute exercise and trained groups showed a higher (*p* < 0.05) phagocytic activity than those isolated from sedentary control groups, in both healthy lean (Figure 3A) and HFD obese (Figure 3B) animals. The highest values of phagocytic activity were determined in the group of acute exercise animals, thus physiologically compensating for the lowest values in the percentage of macrophages with phagocytic capacity in these groups (Table 3). Although without statistically significant differences, only macrophages from both lean (*p* < 0.06; Figure 4A) and obese (*p* < 0.07; Figure 4B) trained animals showed augmented microbicide capacity.

When analysing the influence of exercise on the effect of terbutaline, it was observed that acute exercise did not affect the inhibitory capacity (*p* < 0.05) of β2-adrenergic activation on the phagocytic (Figure 2A,B,C) and microbicide activities (Figure 3A,B,C) determined in sedentary groups, with a similar behaviour in obese and healthy lean animals. However, this terbutaline-induced decline in phagocytic and microbicide activities was abolished in macrophages from trained animals, even determining a stimulatory effect (*p* < 0.05 with respect to sedentary ones), particularly in lean mice (Figure 3A) and when the results are expressed in percentage change from the baseline (Figure 2C and Figure 3C). With little differences, similar results from an immunophysiological point of view were determined when evaluating the percentage of macrophages with phagocytic and microbicidal capacities (Table 3 and Table 4).

### 3.4. Effect of Obesity and Exercise in the β2-Adrenergic Receptor Expression in Peritoneal Macrophages

**Figure 5:**
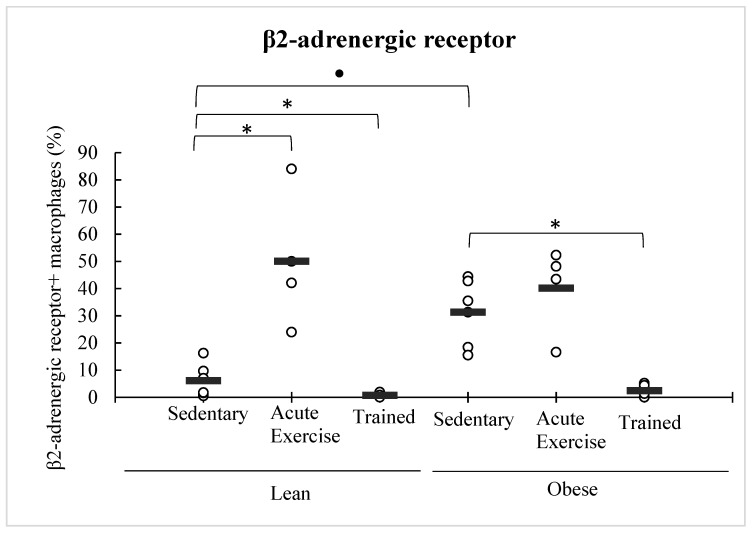
Effect of exercise on the expression of β2-adrenergic receptor in peritoneal macrophages from lean and obese mice (sedentary, acute exercise, and trained mice). Each dot represents the individual values obtained in duplicate in each animal, and the horizontal line represents the mean. * *p* < 0.05 with respect to the corresponding sedentary group; ● *p* < 0.05 with respect to the lean sedentary group.

Obese animals expressed higher levels of β2-AR in peritoneal macrophages than the lean control group. Macrophages from animals performing acute exercise, particularly lean ones (*p* < 0.05), increased the expression of this receptor (as can be observed in the dot graph, this increase in macrophages expressing β2-AR was not statistically significant in the obese mice group owing to an outlier value, atypically low). However, on the contrary, macrophages from trained animals showed a significant decline (*p* < 0.05 with respect to sedentary animals) in the β2-AR expression, in both lean and obese mice (Figure 5).

## 4. Discussion

It is well-known that a pro-inflammatory status together with a dysregulated innate response underlies obesity, and that the beneficial effect of exercise on this pathophysiological condition is importantly mediated by its anti-inflammatory properties via β2 adrenergic regulation [8,9,18]. However, to the best of our knowledge, nothing is known about the role of exercise on the effect of β2-adrenergic activation of macrophages on the performance of their innate immune activity against pathogens. This is very important, because a synergistic potentiation of inhibitory effects by inappropriate exercise practice could promote an undesired immunocompromised status.

In the present investigation, we studied these innate responses (phagocytosis and microbicidal capacities) in macrophages from an animal model of HFD-induced obesity after activation of β2-AR by terbutaline. As expected (confirming the validity of this animal model of obesity-induced metabolic impairment), obese mice showed higher levels of glucose, triglycerides, and cholesterol than the lean control group, and trained obese animals (but not mice performing one session of acute exercise) showed improved dyslipidemia values. In the same animal model of HFD-induced obesity, a recent investigation from our group showed a pro-inflammatory status mediated by the synthesis of inflammatory cytokines by monocytes [8]. However, although it has been reported that mice with HFD-induced obesity present greater susceptibility to infection [29,30], and that human and animal models of obesity have been associated with impaired microbicidal activity [19], the results obtained in the present investigation only found a trend (without statistical differences) towards a lower phagocytic and microbicide activities against *E. coli* of peritoneal macrophages from obese mice with respect to lean, healthy ones. In previous investigations, we observed a lower microbicidal activity against *C. albicans* of peritoneal macrophages from genetically obese rats [15,19]. Recent research has found that only phagocytes from adipose tissue of obese rats (but not those from peritoneum or circulation) show impaired phagocytic activity. Then, it seems that obesity-induced changes in the innate response of phagocytes depend not only on the animal model of obesity, but also on the tissue and differentiated status of these cells. In any case, it is accepted that macrophages play an important role in both the pathophysiology of obesity and in the potential immunosuppression-induced high susceptibility to infections in obese individuals. In this context, both non-pharmacological anti-inflammatory strategies such as exercise [18] and pharmacological ones such as β2-AR agonists [31,32] aimed at regulating the inflammatory activity of macrophages in obesity and diabetic complications, should avoid, if optimal, a potential immunocompromised status of obese individuals against pathogen challenge, particularly when exercise and pharmacological strategies are combined [9,19].

As expected, the selective β2-adrenergic agonist terbutaline decreased the phagocytic and microbicide activities of macrophages (the effect was blocked by the β2-adrenergic antagonist butaxamine); this inhibitory effect was not modified by obesity. Other results have also indicated that the mobility of macrophages (a previous stage of the phagocytic process) is also reduced by β2-adrenergic stimulation. Nevertheless, previous results from our laboratory showed that NA can mediate some of the effects of exercise in stimulating the phagocytic process of peritoneal macrophages [6]. However, this stimulation of the phagocytic process of macrophages by NA needs the participation of α-AR [33,34], which seems to clearly indicate that inhibitory effects of the phagocytic and microbicide activity of macrophages are selectively mediated by β-AR. The questions now can be as follows: Can exercise affect the innate response of macrophages in the same way in healthy lean and obese animals? Can exercise affect the β2-adrenergic regulation of the innate response of macrophages? And in the same way in lean and obese animals?

With respect to the first question, as expected, obesity did not significantly affect the effect of exercise on the innate response mediated by macrophages. Thus, both acute and regular exercise stimulate the phagocytic capacity of peritoneal macrophages in both lean and obese animals, confirming previous results from our group in different animal models [35,36,37], including obese ones [15,19]. This effect was mainly the result of an increase of phagocytic activity, but not in the percentage of macrophages with phagocytic capacity, particularly in the group of acute exercise in which a lower percentage of phagocytosis was compensated by a drastic increase in the phagocytic activity, with a similar behavior between healthy lean and obese mice. However, no significant changes were determined when evaluating the microbicide capacity of peritoneal macrophages from the exercised animal groups (both acute and trained, and both obese and lean) with respect to sedentary control groups. Only a strong tendency towards a greater capacity was observed in the trained animals, also confirming that regular exercise can exert better adaptations to improve the killing of pathogens by innate immune cells [4,38].

With respect to the second and third questions, the inhibitory effect of terbutaline observed in the lean and obese sedentary groups was also observed in the acute exercise groups, affecting both phagocytic and microbicide capacities of peritoneal macrophages. However, it is very interesting to note that the terbutaline-induced decrease in the phagocytic and microbicide capacities of macrophages in sedentary and acute exercise animals was not observed in the group of mice subjected to the program of regular exercise (trained mice). On the contrary, the results indicated that, in general, β2-adrenergic stimulation increased the innate response of macrophages, potentiating the effect of regular exercise. Thus, regular exercise could avoid a potential immunocompromised status against pathogen challenge during the treatment with β2-adrenergic agonists (focused on preventing “sterile inflammation”), which has been recommended for inhibiting the diabetes-induced inflammatory activation of monocytes/macrophages [32], as we have recently reported as effective in this context in the same animal model of HFD-induced obesity [8]. Although obesity did not alter this response with respect to phagocytosis, this pathophysiological condition makes this adaptation induced by regular exercise difficult with respect to the microbicide capacity, as the trained obese group did not show a terbutaline-induced stimulation for killing *E. coli*, as was observed in lean animals. To the best of our knowledge, these are the first published results regarding the role of obesity, and of exercise on this condition, on the β2-adrenergic regulation of the innate response (phagocytosis and microbicide activity) of macrophages; this is the reason we cannot discuss the results with respect to other previous publications.

Nevertheless, previous results have been published in the context of obesity and exercise-induced variations in the levels of endogenous adrenergic agonists, such as NA, and in the expression of β2-AR in obesity [7,18,19,39]. Underexpression of β2-AR on peripheral blood mononuclear cells (PBMC) (as measured by mRNA levels) has been linked with the inflammatory status in obesity [39]. However, the results from the present investigation show that peritoneal macrophages from obese mice expressed more β2-AR than those from lean mice (as measured by flow cytometry in the membrane surface). In this context, high levels of NA in obese animals [7] together with higher β2-AR expression in peritoneal macrophages could be contributing to a lower innate immune response capacity of phagocytic cells against pathogen challenge. However, no available information is clear on the effect of exercise on the surface expression of β2-AR in the membrane of macrophages. In the context of exercise, although controversial, only a couple of studies on PBMC and monocytes [40,41] have reported an upregulation of this receptor after an acute bout of exercise, in accordance with the results of the present investigation. Nevertheless, while macrophages from animals performing acute exercise showed higher expression levels of β2-AR with respect to sedentary ones, the trained groups showed a decreased expression of this receptor in the surface of peritoneal macrophages with respect to sedentary mice. This was observed in both healthy lean and obese animals. It has been speculated that decreased levels of β2-AR expression in circulating monocytes from obese individuals could be linked to the infiltrating capacity of these cells into the adipose tissue [39]. It seems clear that the obesity and exercise-induced variations in the expression of β2-AR depend not only on immune cell subpopulations, but also on their location, probably being different in circulating monocytes and in resident macrophages in different tissues. Thus, it could be plausible to speculate that non-infiltrated peritoneal macrophages in obese individuals (the macrophage population evaluated in the present investigation) are those with a higher level of β2-AR expression, contrary to what could be hypothesized for macrophages with a pro-inflammatory infiltrating profile. In any case, if we accept that the expression of these adrenoreceptors is linked to a greater anti-inflammatory status, their expression levels in peritoneal macrophages could not be considered as a signature of the inflammatory status in obesity, as has been recently proposed for monocytes, although based on the results of total PBMC [39].

In addition, taking into consideration these results, the effects of terbutaline on the phagocytic and microbicidal capacity of macrophages appear not to depend exclusively on β2-AR expression levels, as the higher levels of expression in macrophages from obese animals do not correspond to a greater inhibition of terbutaline in this group. Still, it should not be ruled out that the greater expression of β2-AR is a physiological adaptation in obese animals to achieve similar levels of response to agonist stimulation as healthy lean animals. In fact, and taking into account that obesity is also considered as a model of premature aging [42], the above mentioned interpretation would be in agreement with previous results showing that macrophages from old mice need higher concentrations of NA than those from lean ones to get the same optimal innate responses [20,43]. On the other hand, the decrease in the surface expression of β2-AR after the protocol of regular exercise could explain, at least partially, the observed absence of an inhibitory effect of terbutaline in macrophages from the trained obese and lean groups.

It is accepted that β2-AR are the most relevant and crucial adrenoreceptors expressed in the immune cells in mediating the effect of catecholamines, particularly the anti-inflammatory effects [44]. The results of the present and other recent investigations have shown the complexity of β2-adrenergic regulation of innate/inflammatory cells, particularly in obesity and exercise in this condition, as has been concluded in a recent review [9]. Further studies are clearly needed to delve into the role of β2-AR expression and their sensitivity in response to agonists in different physiological and pathophysiological (such as obesity) circumstances. This is particularly important, considering previous results showing a different response in the inflammatory effects of terbutaline between lean and obese animals [8], and that this pharmacologically-induced anti-inflammatory effect could immunocompromise the innate response in obesity. In this context, regular exercise can ameliorate this potential innate deficiency. Even taking into account the difficulty of obtaining human tissue macrophages, further studies in human subjects seem to also be necessary in order to assure the prescription of optimal exercise programs in obese patients. Macrophages from the adipose stromal vascular fraction from human lipoaspirates could represent a good model for this purpose.

## 5. Conclusions

Finally, in our opinion, the main and physiologically relevant conclusions of the present investigation are as follows:

- The β2-AR agonist terbutaline decreases the phagocytic and microbicide capacities of peritoneal macrophages in sedentary animals, and obesity does not alter this β2-AR neuroimmunomodulation of macrophages.
- Regular exercise causes an adaptation in the response of peritoneal macrophages to the β2-AR agonist terbutaline, by inducing a stimulatory effect in the innate response of macrophages. Obesity seems to hinder this immunophysiological adaptation to regular exercise. This effect is contrary to those produced in both sedentary and acute exercise lean and obese animals.

These results can contribute to the development of optimal new combinations of pharmacological (such as terbutaline) and non-pharmacological (such as regular exercise) interventions for the treatment of inflammation and inflammatory comorbidities in obesity.

## Figures and Tables

**Table 1:** Body weight, dietary intake, and fasting blood glucose and lipid profile in lean and obese mice, without (sedentary) and with exercise, both acute and habitual (trained).

	Lean			Obese		
	Sedentary	Acute Exercise	Trained	Sedentary	Acute Exercise	Trained
Body Weight (g)	29.3 ± 1.2	30.5 ± 2	25.6 ± 1 *	42.3 ± 1.15 •	39.8 ± 2	36.0 ± 3.0 *
Dietary Intake (g/day)	4.0 ± 0.1	4.2 ± 0.1	4.1 ± 0.1	2.7 ± 0.1 •	2.6 ± 0.1	2.5 ± 0.03 *
Energy Intake (Kj/day)	55.32 ± 3.1	58.2 ± 1.4	56.6 ± 0.9	62.01 ± 2.7 •	60.5 ± 1.9	57.6 ± 0.7 *
Glucose (mg/dL)	218.9 ± 13.2	17 4± 32 *	196.4 ± 25	311 ± 31 •	222.7 ± 23 *	282.5 ± 28
Cholesterol (mg/dL)						
Total Cholesterol	103.7 ± 2.2	<99 †	106.7 ± 3	172.7 ± 19 •	175 ± 42	178.1 ± 25
HDL-C	42.1 ± 2.9		51.7 ± 4 *	59.7 ± 5.7 •	55.5 ± 3	75.3 ± 4 *
cLDL-C	50.7 ± 3.5	88.0 ± 0	39.4 ± 2 *	88.8 ± 16 •	78 ± 12	38.5 ± 1 *
Triglycerides (mg/dL)	86.8 ± 1.9	88.0 ± 0	76.6 ± 1 *	91.5 ± 2 •	98.7 ± 7	80 ± 1 *

Each value represents the mean ± SEM of the determinations (one per independent animal) in duplicate. * *p* < 0.05 with respect to the corresponding sedentary group value; • *p* < 0.05 with respect to the lean sedentary group values. HDL-C, high-density lipoprotein cholesterol; cLDL-C, calculated low-density lipoprotein cholesterol; † below limit of detection.

**Table 2:** Effect of the β2-adrenergic agonist terbutaline and β2-adrenergic antagonist butaxamine on the phagocytic percentage and microbicide percentage of peritoneal macrophages from lean and obese mice.

		Control	Terbutaline	Terbutaline + Butaxamine
Phagocytic percentage	lean	85.3 ± 4	79.6 ± 3 *	80.2 ± 4
	obese	77.6 ± 5	71.9 ± 3 *	78.3 ± 4 •
Microbicide percentage	lean	65.8 ± 7	64.9 ± 7	64.2 ± 8
	obese	73.1 ± 8	67.6 ± 8 *	67.7 ± 8

Each value represents the mean ± SEM of independent assays performed in duplicate. * *p* < 0.05 with respect to the corresponding control value, in the absence of terbutaline and butaxamine; • *p* < 0.05 with respect to the corresponding terbutaline value.

**Table 3:** Effect of the β2-adrenergic agonist terbutaline on the phagocytic percentage and microbicide percentage of peritoneal macrophages from lean and obese mice (sedentary, acute exercise, and trained mice).

		Sedentary		Acute Exercise		Trained	
		Control	Terbutaline	Control	Terbutaline	Control	Terbutaline
Phagocytic percentage	lean	85.3 ± 4	79.6 ± 3 *	61.9 ± 5 •	57.4 ± 6 *	68.6 ± 11	67.9 ± 10
	obese	77.6 ± 5	71.9 ± 3 *	64.9 ± 1 •	61.6 ± 2 *	65.4 ± 1	65.7 ± 11
Microbicide percentage	lean	65.8 ± 7	64.9 ± 7	57.7 ± 2	54.3 ± 15	71.7 ± 5	71.8 ± 6
	obese	73.1 ± 8	67.6 ± 8 *	74.8 ± 7	65.1 ± 5 *	62.1 ± 6	61.9 ± 7

Each value represents the mean ± SEM of independent assays performed in duplicate. * *p* < 0.05 with respect to the corresponding control value, in the absence of terbutaline; • *p* < 0.05 with respect to the sedentary control group.

**Table 4:** Influence of exercise on the relative effect of the β2-adrenergic agonist terbutaline on the phagocytic and microbicide percentages of peritoneal macrophages from lean and obese mice (sedentary, acute exercise, and trained mice) (giving 100 to control values in the absence of terbutaline).

		Without Terbutaline	With Terbutaline		
			Sedentary	Acute Exercise	Trained
Phagocytic percentage	lean	100	93.5 ± 2	92.4 ± 2	99.1 ± 3 •
	obese	100	93.3 ± 3	94.9 ± 3	100.5 ± 2 •
Microbicide percentage	lean	100	98.2 ± 4	93.3 ± 3	100.5 ± 4
	obese	100	91.1 ± 4	87.5 ± 4	99.2 ± 3 •

Each value represents the mean ± SEM of independent assays performed in duplicate. • *p* < 0.05 with respect to the corresponding sedentary group.
